# Measuring the Effects of Sharing Mobile Health Data During Diabetes Consultations: Protocol for a Mixed Method Study

**DOI:** 10.2196/16657

**Published:** 2020-02-10

**Authors:** Meghan Bradway, Alain Giordanengo, Ragnar Joakimsen, Anne Helen Hansen, Astrid Grøttland, Gunnar Hartvigsen, Pietro Randine, Eirik Årsand

**Affiliations:** 1 Norwegian Center for E-health Research University Hospital of North Norway Tromsø Norway; 2 Department of Clinical Medicine University of Tromsø-The Arctic University of Norway Tromsø Norway; 3 Department of Computer Science University of Tromsø-The Arctic University of Norway Tromsø Norway; 4 Department of Internal Medicine University Hospital of North Norway Tromsø Norway; 5 Department of Community Medicine University of Tromsø-The Arctic University of Norway Tromsø Norway; 6 Centre for Quality Improvement and Development University Hospital of North Norway Tromsø Norway; 7 Faculty of Health and Sport Science University of Agder Grimstad Norway

**Keywords:** diabetes, patient-gathered data, mHealth, data sharing, therapeutic relationship

## Abstract

**Background:**

There is rising demand for health care’s limited resources. Mobile health (mHealth) could be a solution, especially for those with chronic illnesses such as diabetes. mHealth can increases patients’ options to self-manage their health, improving their health knowledge, engagement, and capacity to contribute to their own care decisions. However, there are few solutions for sharing and presenting patients’ mHealth data with health care providers (HCPs) in a mutually understandable way, which limits the potential of shared decision making.

**Objective:**

Through a six-month mixed method feasibility study in Norway, we aim to explore the impacts that a system for sharing patient-gathered data from mHealth devices has on patients and HCPs during diabetes consultations.

**Methods:**

Patients with diabetes will be recruited through their HCPs. Participants will use the Diabetes Diary mobile phone app to register and review diabetes self-management data and share these data during diabetes consultations using the FullFlow data-sharing system. The primary outcome is the feasibility of the system, which includes HCP impressions and expectations (prestudy survey), usability (System Usability Scale), functionalities used and data shared during consultations, and study-end focus group meetings. Secondary outcomes include a change in the therapeutic relationship, patient empowerment and wellness, health parameters (HbA_1c_ and blood pressure), and the patients’ own app-registered health measures (blood glucose, medication, physical activity, diet, and weight). We will compare measures taken at baseline and at six months, as well as data continuously gathered from the app. Analysis will aim to explain which measures have changed and how and why they have changed during the intervention.

**Results:**

The Full Flow project is funded for 2016 to 2020 by the Research Council of Norway (number 247974/O70). We approached 14 general practitioner clinics (expecting to recruit 1-2 general practitioners per clinic) and two hospitals (expecting to recruit 2-3 nurses per hospital). By recruiting through the HCPs, we expect to recruit 74 patients with type 2 and 33 patients with type 1 diabetes. Between November 2018 and July 2019, we recruited eight patients and 15 HCPs. During 2020, we aim to analyze and publish the results of the collected data from our patient and HCP participants.

**Conclusions:**

We expect to better understand what is needed to be able to share data. This includes potential benefits that sharing patient-gathered data during consultations will have on patients and HCPs, both individually and together. By measuring these impacts, we will be able to present the possibilities and challenges related to a system for sharing mHealth data for future interventions and practice. Results will also demonstrate what needs to be done to make this collaboration between HCPs and patients successful and subsequently further improve patients’ health and engagement in their care.

**International Registered Report Identifier (IRRID):**

DERR1-10.2196/16657

## Introduction

### How Patient Mobile Health Apps Are Changing Consultations

Mobile health (mHealth) technologies originally were designed for and used by patients to better understand and self-manage their health. For those with diabetes, this means tracking and understanding how many different factors, such as diet, exercise, and medication, affect their blood glucose levels. As a result of collecting and reviewing these data, patients are more empowered and knowledgeable, eager to take control and responsibility of their daily health, and more knowledgeable patients are able to better understand how their actions affect their diabetes health. Some information and subsequent decisions are more evident than others after reviewing their data. In other words, patients can only understand or explain a portion of the data that they collect without the medical expertise of health care providers (HCPs) to contextualize these data with known disease processes. Patients have begun to bring these data from their mHealth technologies to their health care consultations, hoping that the HCPs can provide explanations for the results seen in their gathered data [1].

### Mobile Health Data Sharing

The expected benefits of mHealth integration and data sharing are to decrease health care costs, increase patient engagement and aid options, and improve clinical outcomes [2,3]. However, HCPs have traditionally relied on scientifically proven, professionally collected clinical data, such as laboratory test results and biological measures taken at consultations, to understand the patient’s health status. There is evidence that by using these data to inform a clinical recommendation, HCPs can be confident that they have provided a relatively accurate diagnosis and that their treatment will produce a known clinical outcome [4]. Ideally, presenting app-collected data to HCPs would provide a greater understanding of the patient’s situation. However, the data have not been collected, structured, or validated in relation to disease status like traditional laboratory data. The presentation and structure of the data (ie, dozens or hundreds of data entries), including many different types of data from a variety of different mHealth technologies, is a challenge to relate to from the HCPs’ perspective.

Further, HCPs aim to use medical data in a slightly different way than patients use their patient-gathered data. In other words, each wants to know different things. The patient wants to know if their daily decisions are having a positive effect on their disease management, and the HCPs want to know how their clinical recommendations and medications are affecting the disease status. These priorities are complementary; as part of daily self-management, diabetes patients need to observe, understand, and respond to fluctuations in their blood glucose [5], often instantly for those with type 1 diabetes. The focus of HCPs is on the progress or trend to determine if a treatment modality or approach is a practical choice for that patient in the long run [6,7]. Therefore, for mHealth data sharing to be useful for patients and HCPs, the information should be presented in a way that both can understand, discuss, and use together to determine how best to maintain or improve treatment and self-management strategies. This is an example of shared decision making.

### The Potential of Shared Decision Making

Shared decision making describes the communication and health care decisions made between patients and their HCPs [8]. When used in such a way, shared decision making is key to successful therapeutic relationships—those between patients and their HCPs—and, ultimately, patients’ adherence and achievement of treatment aims [8]. Several studies have demonstrated that patients’ improvement in HbA_1c_ (glycated hemoglobin) and perceived diabetes competence are associated with a medical environment where clinicians encourage patients’ autonomy [9,10].

### Sharing Mobile Health Data Enables Shared Decision Making

With mHealth, individuals have been presented with the opportunity to bring patient-relevant data to the conversation during consultations, as opposed to relying on only patient memory of their self-management and clinical test results. In doing so, true shared decision making between the HCP and patient is not only possible but necessary to effectively support and validate patient decisions in their self-management. For example, a patient may collect diet or exercise data that could explain fluctuations in clinical test results, such as lipid levels or imbalances between insulin and blood glucose levels during those periods. Patient-gathered data could even bring to light challenges that the patient faces in their self-management that are not evident from clinical test results, such as dangerous nightly hypoglycemic events. The result of bringing such information to the consultation is, for example, that the patient could provide concrete evidence of their challenges and self-management activities, with specific questions that would improve their understanding and ability to self-manage. Then the HCP could explain why adverse outcomes are occurring and give patient-tailored guidance about how to better deal with such situations in the future. Therefore, patient-gathered data from these devices could strengthen patient-clinician collaboration in tailored diabetes treatment.

### How to Approach Mobile Health Intervention Research

The purpose of health intervention research is to develop and test the ability of such things as a new device, system, or service to improve patient health outcomes or experiences. To develop a solution that facilitates shared decision making using mHealth data, one must consider two main questions: (1) how to effectively present the mHealth-gathered data during consultations between patients and HCPs, and (2) how to promote conversation about the patient-gathered data in a way that leads to shared decision making. The goal of testing such a solution is typically to determine if a system can successfully convert patient-gathered data to a form that is understandable and useful for patients and HCPs and shows that the use of the data can produce positive clinical or experiential outcomes.

### The Proposed Solution

To assess how mHealth data sharing comprehensively affects patients, their health outcomes, HCPs, and their therapeutic relationship, we must first have a suitable data-gathering and data-sharing platform that can facilitate and validate this new situation. As the data-gathering platform, we use a mobile phone app, the Diabetes Diary, which has been tested in several studies [11-14]. In the Full Flow of Health Data Between Patients and Health Care Systems project, we aim to design, develop, and test a system for sharing patient-gathered mHealth data with HCPs during diabetes consultations by iteratively involving both patients and providers throughout the research activities [15].

**Figure 1:**
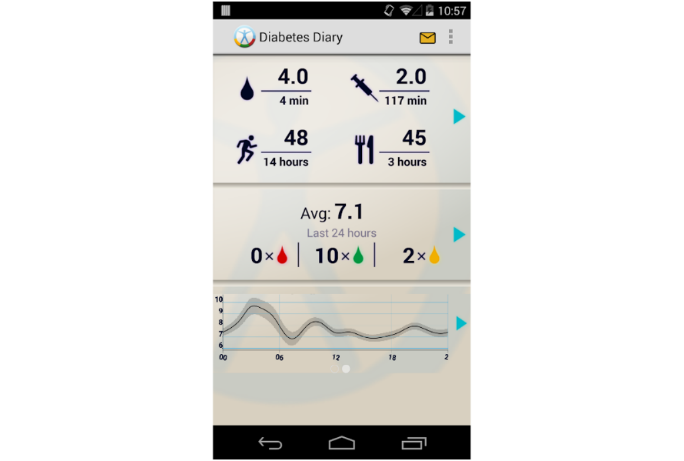
Home screen of the patient-operated Diabetes Diary app (English version).

The Diabetes Diary app is a research tool that allows patient participants to register their self-gathered health measurements (eg, blood glucose and physical activity) and review previously registered data either as a summary or list (Figure 1). Patients then have the option to select the data they want to share with their HCPs, which is then displayed via the FullFlow System, a platform for sharing and presenting patient data.

**Figure 2:**
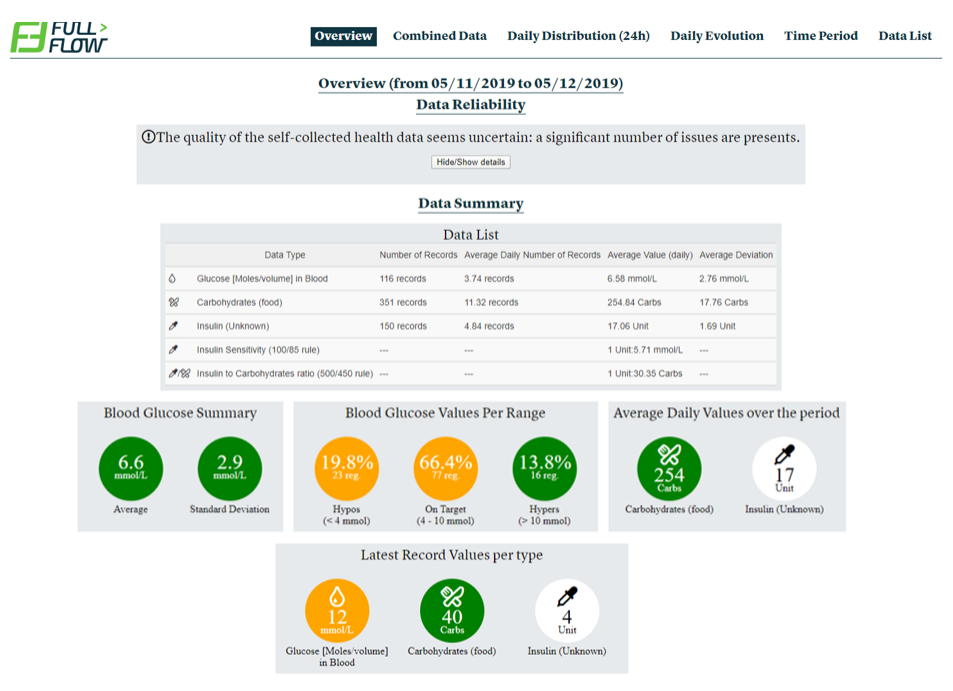
The FullFlow System’s Web-based home screen (English version).

The FullFlow System’s Web interface allows both patients and providers to view together selected summaries and preliminary information about the set of shared data. This system also allows users to choose the summary forms to view, which is intended to be guided by the information about the patient’s progress on their goals, measurements, and identified areas of possible concern illustrated on the home screen (Figure 2). The development details and initial clinical testing of the data-sharing system are described elsewhere [16,17].

### Study Aims and Objectives

A working version of the system was developed in 2018 [17-19]. We now aim to comprehensively measure its impacts on patients and providers and its role in encouraging patient-provider collaboration in diabetes care. Further, by using diverse measures (mixed methods) based on different research fields (eg, psychology, medicine, and technology), we can better understand which impacts mHealth can have on health care services.

The overall objective is to understand and test the effects of using a data-sharing system (exemplified by the FullFlow System developed in-house [17,20]) for patient-gathered mHealth data and the Diabetes Diary mobile phone app. We hypothesize that sharing such data, in the form of mutually relevant information, will enable patients and HCPs together to generate more tailored and concrete self-management recommendations for patients. This protocol includes a description and justification behind why the selected measures, evaluation methods, and study implementation methods were chosen.

## Methods

### Study Design

**Figure 3:**
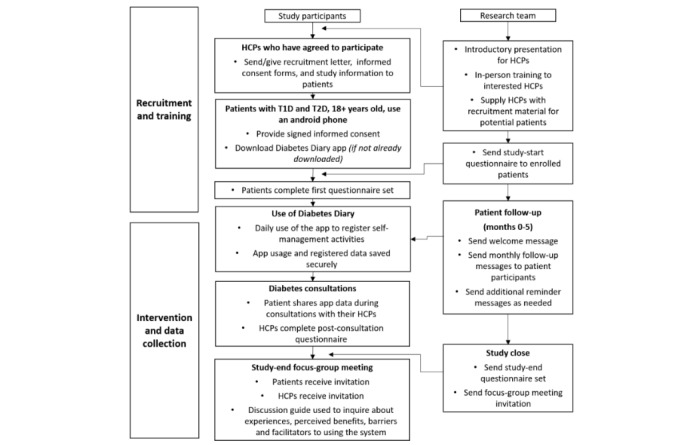
Study design flowchart. HCP: health care provider; T1D: type 1 diabetes; T2D: type 2 diabetes.

This protocol describes a six-month mixed method study, which is part of the larger Full Flow project [15], in which the FullFlow data-sharing system is used to enable the sharing of patient-gathered mHealth data during diabetes consultations. The design of both the data-sharing system itself and mixed method study structure (Figure 3) are based on developmental studies and activities within the Full Flow project, described elsewhere [16,18,19,21,22].

Traditionally, health studies report only the pre- and posteffects of interventions, perhaps with some participant-recalled experiences. However, human memory is prone to forgetfulness and mistakes. Using mHealth technology that can provide real-time recording of information about what patients did and how their health responded to their self-management is an invaluable resource for health studies. Therefore, in the described study, we include a comprehensive set of measures that take advantage of the reliability of clinical measures and standardized questionnaires with the record of how patients performed their self-management between consultations (see Table 1). In doing so, we aim to understand not only the pre- and posteffects of using such a system but also how patients performed their self-management between consultations.

**Table 1:** List of data types, their sources, and purpose for measurement.

Data collection tools^a^		Purpose: to measure...	When collected
**Primary outcomes**			
	Prestudy survey to HCPs^b^	HCPs’ first impressions of the system and their expectations	Before study start
	Postconsultation questionnaire	Functions of the system used, HbA_1c_^c^, and blood pressure of patients	After each consultation
	Data displayed by the FullFlow System	What patients chose to share during consultations	At each consultation
	Study-end focus group meetings^d^	Experiences, perceived benefits, barriers to, and facilitators for using the system	After study end
	System Usability Scale [23]	Usability of the system for patients	After study end
**Secondary outcomes**			
	Diabetes Empowerment Scale [24]	Patient engagement (ability)	Before and after the study
	WHO-5 wellness [25]	Patient engagement (likelihood)	Before and after the study
	Health Care Climate Questionnaire [26]	Therapeutic relationship	Before and after the study
	Patient-registered health data (app)	Patients’ self-measured health parameters: blood glucose, weight, diet, physical activity, and medication	Continuously throughout the study
	App usage logs	Patients’ interactions with the Diabetes Diary app	Continuously throughout the study

^a^ Norwegian versions of all questionnaires will be used. The five-question World Health Organization Wellness Index (WHO-5) is the only Norwegian version of a questionnaire to be officially validated [27].

^b^HCP: health care provider.

^c^HbA_1c_: glycated hemoglobin.

^d^Focus group sessions will be held in Norwegian, audio-recorded, transcribed, cleared of all identifiable information, and translated into English for analysis.

### Online Study Administration and Management

The online study management platform provides a real-time overview of participants’ progress through the study. The platform allows study administrators to deliver recruitment material and collect informed consent electronically. After it is confirmed that the patient has downloaded the app and entered the code, we can collect their data. Each participant is assigned an anonymous user ID, which is not directly linkable to the user’s personal information (eg, personal email, sensitive personal information) that is stored elsewhere. Electronic questionnaires and direct follow-up messages can then be sent to these user IDs, directly to the app, and registered personal email. This direct channel with the app also allows the platform to collect both registered measurements and usage log data from the app. Preliminary and summative analysis is accessible via the system as well to identify data gaps, such as possible technology challenges that participants are experiencing that study administrators can efficiently respond with follow-up messages when necessary.

### Study Population

We recruited general practitioners (GPs), diabetes nurses, and individuals diagnosed with either type 1, type 2, or other types of diabetes in the Troms and Finnmark region of Norway between October 2018 and July 2019. Inclusion to participate as a health care provider required that they had the ability and willingness to use the FullFlow data-sharing system during their consultation setting, which required an internet connection and a Web browser on their office computer. Persons with diabetes who were older than 18 years were eligible to participate. Inclusion required that they have a mobile phone with an Android operating system through which the Diabetes Diary app could be downloaded and used for data collection. Participants had to be willing to use the app to gather and share data during consultations, and to consider participation in a study-end focus group meeting. No restrictions were placed on applicants’ disease duration or HbA_1c_ level. Exclusion criteria included any mental or physical illness that interfered with their ability to fulfill study expectations.

### Recruitment and Training

#### Health Care Personnel

We require sets of patients and their health care professionals to agree to participate together; therefore, we will first approach diabetes nurses and endocrinologists through our research team’s current network, including the University Hospital of North Norway and Hammerfest Hospital. A member of our research team will identify potential GP participants and cold-call them directly. Emails will also be used to request in-person recruitment meetings. Two representatives of our research team will give a brief lunch presentation to HCP offices accepting such meetings. For those interested in participating in the study, we will schedule one-hour training sessions to demonstrate the FullFlow data-sharing system in more practical detail on the HCP’s own computer. The HCPs will be asked to complete a brief survey about their perceptions of the presented FullFlow System after these in-person training sessions.

As GP offices in Norway do not commonly have agreements or contracts with local or national research projects, we will provide additional compensation for the time taken outside of their regular work schedules for the training sessions for each patient enrolled and for any additional time spent on the study, such as study-end focus group meetings. These will follow standard reimbursement schemes for health care professionals in Norway.

#### Patients

When needed, we will also assist HCPs in identifying potential participants from their diabetes patient lists. We will provide both electronic and paper copies of the patient recruitment materials. HCPs will provide patients the recruitment letters and study information in-person during consultations, or they will mail the letters to those not scheduled to meet for consultation shortly after. Patients will be instructed to contact us if they are interested in enrolling in the study. Patient recruitment letters will contain a link to the study webpage where interested patients will be able to read and sign the informed consent form electronically (Multimedia Appendix 1). Patients who have not already downloaded the tailored version of the Diabetes Diary app, including an associated website and user guide [28], will be requested to do so to participate. We will also inform patient participants of their right to withdraw their data or participation from the study at any time. Patients will be reimbursed for travel and consultations if the meetings are scheduled in addition to their usual care.

All participants will be encouraged to participate in the study-end workshop. The participants are informed that technical support will be available via email or by visiting our office. Patient recruitment ended on July 1, 2019.

#### Sample Size

We plan to approach 14 GP clinics, with an estimated one to two interested GPs in each clinic, and two hospitals, with one to three nurses and one endocrinologist in each.

The GPs in the Troms and Finnmark regions of Norway have listed 1000 to 1500 patients [29]. The prevalence of type 2 diabetes is 4.7% [30]; therefore, our recruitment pool is expected to be 1234 patients with type 2. The average expected response rate is 15% (range 10% to 20%), and approximately 40% of these patients are estimated to meet the inclusion criteria. Therefore, we expect to recruit 74 patients with type 2 diabetes.

There are 511 patients with type 1 diabetes registered at University Hospital of Northern Norway (UNN) Tromsø and 62 registered at Hammerfest Hospital in the Norwegian Diabetes Registry for Adults [31]. With the same estimated response rate of 15% and 40% of these meeting the inclusion criteria, we estimate to recruit approximately 30 patients from UNN and three patients from Hammerfest Hospital.

### Intervention Description

#### Diabetes Diary Application—Tailored Version

Our research team previously developed a tailored version of the Diabetes Diary app [32,33], which we will provide to all patient participants. We developed the app over several years to act as the research platform for many projects [13,34,35]. The app itself allows patients to tailor the app to their diabetes type and self-management foci, including the ability to register and review the following data types: goals, blood glucose, medication, physical activity, nutrition, and weight.

For the study, both registered measurements and usage log data from this app will be continuously encrypted and transferred to the project’s secure online study management platform [36], which was used during two previous projects [34,37]. However, for consultations, the patient will be able to control the data they share with their health care team via the tested FullFlow System.

#### The FullFlow Data-Sharing System

The FullFlow System will summarize and display information based on the data provided. If patients do not share data, patients and HCPs can plan goals together about which data to collect and discuss during future consultations. We have designed the dynamic, Web-based interface of the FullFlow System to facilitate easy navigation of this information. The FullFlow System will register the data that patients choose to share, which we will then qualitatively analyze after the study. A more detailed description of the FullFlow System itself is described elsewhere [17].

#### Consultations and Self-Management

We will ask that each patient-clinician team schedule at least one consultation by the sixth month of the study related to diabetes treatment. To the best of their ability, HCPs and patient participants should use the FullFlow System during these consultations. HCPs are requested to report the functions that were used, the usefulness of the FullFlow System, and the patients’ HbA_1c_ and blood pressure via a postconsultation questionnaire (requiring three to five minutes).

We will send monthly messages to patients using the online study management platform. These messages will appear both in the participants’ email and the Diabetes Diary app. We detail the scheduled messages (eg, reminders to schedule appointments and register data throughout the study) in Multimedia Appendix 2.

### Data Collection

We will administer questionnaires through LimeSurvey [36,38] and our study management platform. Information about which data was registered in the Diabetes Diary app will be collected continuously through connection to our secure research platform.

We will request patient participants to report the following before study start: age, gender, level of education, disease duration, medication type, and delivery system (eg, pens, pumps, pills). We will also request data, described in the Evaluation Measures section and Table 1, about patients’ self-management habits and perceived health status and challenges that they may have with the self-management of diabetes parameters.

### Evaluation Measures

We chose to include standardized and validated questionnaires where possible, supplemented by measures specific to impressions of the use of the technologies involved. The combination of questionnaires was chosen to limit the number of questions because we are also asking them to track several other factors as part of the intervention on the mobile phone app. Table 1 introduces an overview of the purpose and selection of our data collection tools.

### System Usability

We will assess the usability of the system with three data collection tools: the prestudy survey to HCPs, the System Usability Scale (SUS) [23], and the postconsultation questionnaires. The reason for combining these to measure usability is that responses from each build on one another. In other words, we measure not just overall satisfaction or dissatisfaction with the system, but information about how each pair of patients and providers used the system during each consultation.

The postconsultation questionnaires provide a specific indication of the functions the HCPs and patients chose to use (ie, which characteristics of the system contributed to their use).

### Patient Well-Being and Health

Postconsultation questionnaires will also request that the HCP provide the laboratory values for each patient’s HbA_1c_ and blood pressure. The participants’ own app-registered health data (ie, measured values of blood glucose, administered insulin or other medication, weight, physical activity, diet, and goals) will provide a more continuous illustration of a patient’s self-management foci and health. By comparing these recorded values to the other measures mentioned, we aim to explain how patient self-management habits contribute to measures of health, engagement, and communication with their providers.

The World Health Organization Wellness Index (WHO-5) is a five-question measure of an individual’s subjective health during the previous two weeks using a six-point Likert scale [39]. We chose this measure based on its simplicity, brevity, and ability to cover a diversity of concepts related to well-being.

### Patient Empowerment and Engagement

The Diabetes Empowerment Scale-Short Form (DES-SF) is an eight-item questionnaire that measures an individual’s psychosocial self-efficacy [24]. Self-efficacy refers to a person’s belief in their own ability to perform the activities necessary to achieve a specific level of performance; in this case, those necessary to maintain or improve their diabetes health. Although this is a measure of a person’s belief and not actions, self-efficacy is strongly correlated to an individual’s self-care actions in the case of diabetes [40,41].

The participants’ own app-registered health data are evidence of their real-world self-management habits. Similarly, the interactions with the app (ie, app usage logs) indicate time spent using the app that includes not only time taken to enter values but also the use of other functionalities (eg, reviewing previously recorded materials).

### Therapeutic Relationship

The Health Care Climate Questionnaire (HCCQ) is a six-item measure of patient perception of whether their HCP supports their autonomy [26,42]. In other words, the HCCQ measures the relationship between patients and HCPs. This questionnaire is based on the concepts of self-determination or one’s ability to choose their own actions [43]. The therapeutic relationship supports one’s health self-management and has been shown to significantly contribute to an individual’s health-related outcomes [44]. These concepts describe a collaboration based on mutual contribution to care decisions rather than a patient-provider relationship based on a hierarchy of knowledge and power. In combination with the other questionnaires listed, we can better understand how, and possibly why, a system that encourages communication, initiated by the patient’s choice to share patient-gathered data, affects the patient’s motivation, self-care actions, and health, as described previously.

### Study-End Focus Group Meetings

We have chosen the presented questionnaires to limit “burnout” from answering too many written questions; however, we still expect there to be missing responses. In addition, as this is the first time these measures have been used together in a study for mHealth—to the best of our knowledge—we expect that we will have follow-up questions and clarifications about the patients’ and providers’ responses. Therefore, the study-end focus group meetings will focus on elaborating the participants’ responses from the measures mentioned previously and encouraging the participants to share their experiences and opinions. We also aim to gather more specific input and explanation of the system’s function, use, and suggested improvements.

### Data Analysis

Baseline measures will be described using descriptive statistics. We assume that some variables will differ between participants with different types of diabetes due to the limited size of the study population.

Analysis of responses for all standardized tests will follow the scoring guidelines provided with each measurement tool. Postconsultation questionnaires will be assessed quantitatively and qualitatively, depending on the question type. The transcripts from the study-end focus group meetings will be analyzed using inductive thematic analysis to contextualize the quantitative results. Paired *t* tests will be used to compare all quantitative baseline (0 months) and study-end (6 months) measures. Correlation analysis will be used to assess relationships between quantitative and coded qualitative variables, when possible.

## Results

### Ethical Approval

The protocol, questionnaires, interview guides, recruitment material, and other adjoining study material have been submitted to the Regional Committees for Medical and Health Research Ethics for Northern Norway, who found the study exempt from their purview of approval. Instead, the study was declared and approved by the Personvernombudet (Personal Data Protection Officer) at UNN.

### Funding

This study is part of the first author’s PhD program and has been funded through a larger project, entitled “The Full Flow of Health Data Between Patients and Health Care Systems (2016-2020),” by the Research Council of Norway (number 247974/O70).

### Progress to Date

Recruitment for this six-month study began in October 2018. As of September 2019, we recruited 13 GPs, two diabetes nurses at two hospitals, and eight patients. We expect all results to be collected by March 2020. We will then have results about patient and provider usage of the technologies, collected automatically, as well as their reported experiences. From these, we can identify whether the tested system met their individual needs and potential improvements needed to facilitate collaboration in diabetes care consultations. Results will also include the impact of collaborative use on the patients’ clinically measured data from mHealth tools, as well as their measured health and wellness.

## Discussion

### Collaboration Between Patients and Providers

The described Full Flow mixed method study is the final phase of the Full Flow project. Previous phases of this project engaged individuals with types 1 and 2 diabetes, and a variety of HCPs, in iterative and experience-based activities to design the studied FullFlow data-sharing system. During these initial phases, the concepts of end-user perspective and collaboration between patients and providers, not only in clinical practice but also in research, was emphasized.

Although many studies and commercially available systems involving shared patient-gathered data focus on the provider’s interpretation of the information, we believe that it is not only possible but necessary to encourage more collaboration between and contribution from both parties in mHealth interventions and care practice. Through our choice of methods and measures, we aim to exemplify the importance of accounting for the unique additional needs and opportunities of mHealth in research practice.

### True Shared Decision Making

Shared decision making is described as patients and their HCPs working together to collaborate on the process of making health decisions [45]. However, most interventions describe this process with HCPs taking on the bulk of the decision making [46]. Instead, the patient is queried about their goals and preferences, acting mostly as an information source for the HCP. This lack of a true, equal partnership between patients and HCPs has been cited as mainly due to time constraints and lack of patient engagement or knowledge of their health situation [47]. This highlights the importance of using a patient’s capacity and willingness to contribute to this process.

Today, patients’ use of mHealth and the ability of these technologies to enable collecting and sharing of patient-gathered data make true shared decision making possible. Sharing patient-gathered data allows for a more balanced and patient-initiated process of developing recommendations for self-management (ie, tasks that are performed by the patient on a daily basis).

### Other Measures Are Needed

Within research, we must also adapt to the new situation that mHealth creates. A major challenge of understanding the effects of mHealth interventions is determining which traditional measures are applicable and which others are needed. The World Health Organization (WHO) is an example of several attempts to develop a comprehensive set of information that is needed from mHealth intervention studies. In addition to the traditional usability reports for new health technologies, the mERA (mHealth Evaluation, Reporting and Assessment) checklist also calls for evidence of barriers and facilitators to participants’ access to the intervention (eg, “factors that may limit the users’ ability to use the intervention”) as well as its potential to be implemented into clinical care [48]. The dynamic network of interactions that mHealth represents calls for more than pre- and postintervention measurements. In this context, where patients can use several tools and services continuously in their everyday lives, it is no longer sufficient to merely understand what has changed and by how much [49]. This is our opportunity to invite not just patients and their devices but also their HCPs to participate in considering and understanding the interactions within and outside of clinical practice.

Not only has mHealth provided researchers with a more informed patient, it has also provided us with ways of tracking how they use mHealth (eg, by analyzing app and system’s usage logs) [50]. These allow us to more effectively observe and record patients’ self-management tasks and health measures, their data shared during consultations, and other factors that static questionnaires are not able to collect. One of these factors, which now plays an even more crucial role than before, is the motivation to be more involved in the data collection and sharing process. The relationship—now hopefully, collaboration—between patient and provider is not only something that can change but also something that can play a role in patients’ motivation to engage in their health [51,52]. By including standardized psychological questionnaires with the other measures of patients’ well-being, health, and self-management activity, we can contribute to a better understanding of this relationship. The planned study-end focus groups will allow us to elaborate on why some of these changes are happening and provide insight from all the participants about the context of their decisions.

### Conclusion

In this study, we aim to address and understand the nuances of mHealth. By including measures of what has changed, including how and why, we can begin to more effectively and accurately explore the impacts of mHealth on not only the before and after measures but also events during the intervention itself. In addition to the relevant research communities, the information gained from this study will inform our electronic health record vendor partners and both The Norwegian Directorate of eHealth and overarching Ministry of Health and Care Services [53], which will better prepare Norway, and other countries, when forming future health systems that support mHealth integration.

## Appendix

Multimedia Appendix 1 Informed consent form.

Multimedia Appendix 2 Scheduled follow-up messages to patient participants.

## Abbreviations

GP: general practitioner
HbA_1c_: glycated hemoglobin
HCP: health care provider
UNN: University Hospital of Northern Norway
