# Supplementary material for: Measuring the Effects of Sharing Mobile Health Data During Diabetes Consultations: Protocol for a Mixed Method Study
Source: JMIR Res Protoc. 2020 Feb 10;9(2):e16657. doi: 10.2196/16657 (PMC7055770; doi:10.2196/16657)
Supplement: Multimedia Appendix 1 [file resprot_v9i2e16657_app1.docx]

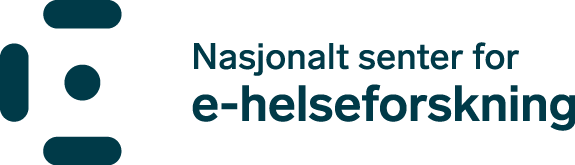


# Forespørsel om deltakelse i forskningsprosjektet

testing av system for Deling av data mellom pasient og helsevesen – Diabetes type 1 og type 2

Dette er et spørsmål til deg om å delta i en forskningsstudie for å teste ut et system for deling av egne innsamlede helsedata fra pasienter til helsevesenet. Du er spurt om å delta siden du har vist interesse for å få informasjon gjennom informasjon utdelt av helsepersonell, sosiale media, eller appen Diabetesdagboka. Studien arrangeres av Nasjonalt senter for e-helseforskning ved Universitetssykehuset Nord-Norge.

## Hva innebærer PROSJEKTET?

Studien innebærer utprøving av en ny måte å møte helsevesenet på ved at du får mulighet til å dele egne helsedata gjennom et nytt system. Dette vil innebære to konsultasjoner med din faste kontakt i helsevesenet omkring diabetes i løpet av et halvt år. I tillegg vil du bli bedt om å delta i en opplæringssesjon i forkant, enten i et fysisk møte dersom du har mulighet til å møte i Tromsø, eller en nettbasert opplæringssesjon om du ikke kan delta fysisk. Vi vil også avholde en avslutningsworkshop der du kan delta for å gi oss tilbakemelding om datadelingssystemet og diskutere erfaringer med hverandre. Til innsamling av data vil du bruke siste versjon av appen «Diabetesdagboka» (du må bruke en Android telefon). Du vil selv kunne avgjøre hvilke data du vil dele med helsepersonell under konsultasjonen fra appen.

Gjennom appen Diabetesdagboka vil du kunne samle inn og holde oversikt over blodsukkerverdier, medisiner, mat/ernæring, fysisk aktivitet, vekt, personlige mål og notater til disse. I opplæringssesjonen vil vi i forskningsgruppen forklare mer om bakgrunnen til studien, og lære deg å bruke appen og datadelingssystemet, samt avklare forventninger for alle parter, inkludert anbefalinger for registrering av data. Du vil ha tilgang til teknisk support gjennom hele studieperioden.

Vi vil be deg svare på spørreskjema etter første og andre konsultasjonen (tar ca. 10 minutter). Ved den første og siste konsultasjonen vil du måle HbA1c og blodtrykk slik at din lege/helsepersonell kan rapportere dette til studien. Lydopptak vil bli gjennomført under avslutningsworkshopen. Helsedata som du samler gjennom appen og bruk av appen vil bli automatisk overført til vår sikre forskningsserver. Dine data vil være kodet og avidentifisert og ikke delt med andre enn de i vårt forskningsgruppe.

Som del av studien vil vi sammenstille opplysningene du samler inn slik at vi kan forstå hvordan bruk av det testede systemet påvirker helseparametre, ditt forhold til helsepersonell og din egenbehandling.

## Mulige fordeler og ulemper

Bruk av appen krever litt ekstra tid hver dag (noen få minutter). Videre vil noen kunne oppleve det som tidkrevende å fylle ut spørreskjema ved møtepunktene i studien. Avhengig av hvor ofte du vanligvis møter din behandler, kan det hende du vil måtte møte hyppigere enn vanlig under studiet. Under konsultasjon vil du ha mulighet til å dele flere egeninnsamlede helsedata enn vanlig, noe som kan endre diskusjonen med helsepersonell. Det er også mulig at din behandler vil ønske å lagre dine data som vedlegg til journalen, og du har rett til å både velge hvilke data du vil dele og om du vil at disse skal lagres. Vi forventer at ved å dele helsedata med din behandler, vil dere kunne ta bedre beslutninger omkring din situasjon sammen. Dersom du og din behandler kan bruke systemet til å bedre forstå din situasjon, kan dere lage bedre og realistiske planer for hvordan oppnå ønsket helsegevinst. Du kan også oppnå å få bedre oversikt og kontroll på din egenbehandling.

## Frivillig deltakelse og mulighet for å trekke sitt samtykke

Det er frivillig å delta i studien. Dersom du ønsker å delta, krysser du av for dette på nederst i denne samtykke- og informasjonsskrivet, og vil deretter få informasjon om oppstart av studien på e-post, inkludert invitasjon til opplæringssesjonen. Du kan når som helst og uten å oppgi noen grunn trekke ditt samtykke til deltagelse. Dette vil ikke få konsekvenser for din videre behandling i helsevesenet. Dersom du trekker deg fra studien, kan du kreve å få slettet innsamlede prøver og opplysninger, med mindre opplysningene allerede er inngått i analyser eller brukt i vitenskapelige publikasjoner. Dersom du senere ønsker å trekke deg eller har spørsmål til studien, kan du kontakte prosjektleder Eirik Årsand, tlf. 992 43592, e-post: [eirik.arsand@ehealthresearch.no](mailto:eirik.arsand@ehealthresearch.no).

## Hva skjer med informasjonen om deg?

Informasjonen som registreres om deg skal kun brukes slik som beskrevet i hensikten med studien. Du har rett til innsyn i hvilke opplysninger som er registrert om deg og rett til å få korrigert eventuelle feil i de opplysningene som er registrert. Dersom du sender inn data, og disse lagres i helsejournalen, så kan du kan be om at disse data slettes igjennom vanlig prosedyre for å slette data fra journalsystemet (via Personvernombudet).

Alle opplysningene vil bli behandlet uten navn og fødselsnummer eller andre direkte gjenkjennende opplysninger. En kode knytter deg til dine opplysninger gjennom en studie-ID liste. Det betyr at opplysningene er avidentifisert. Det er kun autorisert personell knyttet til studien som har adgang til studie-ID listen og som kan finne tilbake til deg. Personidentifiserbare data vil bli slettet i 2024. Det vil ikke være mulig å identifisere deg i resultatene av studien når disse publiseres. Prosjektleder har ansvar for den daglige driften av forskningsprosjektet og at opplysninger om deg blir behandlet på en sikker måte.

## Hva skjer med prøver som blir tatt av deg

Dine blodprøver (HbA1c) og blodtrykksdata, svar på spørreskjema og eventuelle lydopptak av deg vil lagres avidentifisert på en sikker server. Alle opplysningene vil bli behandlet uten navn og fødselsnummer eller andre direkte gjenkjennende opplysninger.

## Forsikring

Eventuelle skader som en følge av studien dekkes i henhold til pasientskadelovens bestemmelser.

## OppfølgingsPROSJEKT

Deltagelse i dette prosjektet innebærer at du kan bli kontaktet igjen med forespørsel om å delta i et oppfølgingsprosjekt.

## Økonomi

Deltagelse i opplæringssesjonen (fysisk oppmøte) og avslutningsworkshopen vil kompenseres med kroner 2000,- (skattepliktig) dersom disse foregår på dagtid. Du vil også få kompensert reise til og fra slike møter etter avtale, billigste reisemåte. Du vil også få kompensert utgifter til ekstra legetimer i de tilfeller der dette kommer i tillegg til dine vanlige møter med helsevesenet.

Prosjektet som denne studien er en del av får økonomisk støtte fra Norges Forskningsråd (programmet IKTPLUSS). Det er ingen interessekonflikter i studien.

## Godkjenning

Prosjektet er godkjent av Personvernombudet ved Universitetssykehuset Nord-Norge [ref. nr. 2018/4027].

# Samtykke til deltakelse i PROSJEKTET

## VED å krysse av i rubrikken nedenfor og trykke «send inn mitt svar» knappen, velger du du å delta i prosjektet, eller ikke. Dersom du velger å delta, vil du motta en e-post med kopi av dette samtykket.

Ja, jeg samtykker til å delta

Nei, jeg vil ikke delta

Send inn mitt svar
