# Supplementary material for: Measuring the Effects of Sharing Mobile Health Data During Diabetes Consultations: Protocol for a Mixed Method Study
Source: JMIR Res Protoc. 2020 Feb 10;9(2):e16657. doi: 10.2196/16657 (PMC7055770; doi:10.2196/16657)
Supplement: Multimedia Appendix 2 [file resprot_v9i2e16657_app2.docx]

**Appendix 2. Follow-up messages sent to the patient-participants of the Full Flow Project’s mixed-method feasibility study**

The purpose of the messages are described in English followed by the messages themselves in Norwegian.

1. If problems with ID entry:

Kjære deltager, vi ser at du ikke har lagt inn deltager-ID for å kunne delta i studien. For å gjøre dette, åpner du appen Diabetesdagboka, gå inn i menyen, velge «Personlige data», trykke på «Delta i forskningsprosjekt» og så skrive inn denne deltager-id: F{{anon_id_without_project_prefix}}. Hvis du har problemer med dette, ikke nøl med å enten ringe oss (992 43 592, sende e-post, eller avtale et møte med oss på vårt kontor i Forskningsparken i Tromsø.

1. Start-up message with education offer, **2 days** after user have entered Project code in the app (Title of message: «Oppstart av diabetesstudien!»): Velkommen som deltager i vår FullFlow-studie! Både hvis du er en ny - eller erfaren bruker av appen Diabetesdagboka, kan du finne ut de ulike måtene å bruke appen på i bruksanvisninga som ligger på studiens web-side: <http://hubro.ehealthresearch.no/fullflow2019/> Dersom du vil ha en praktisk gjennomgang eller mer informasjon, kan du avtale et møte med oss på vårt kontor i Forskningsparken i Tromsø, eller sende oss e-post med spørsmål.
2. Follow-up after **1 month**: (Purpose: we are here to help you, and get to know you app through either the user manual link to the short manual or just ask us) –
   Hei! Vi er glade for at du valgte å delta i vår studie. Det er en knapp måned siden du meldte deg på og vi håper du er kommet i gang med å bruke appen Diabetesdagboka. Husk at du finner både detaljert og en kort bruksanvisning på studiens web-side: <http://hubro.ehealthresearch.no/fullflow2019/>
3. Follow-up after **2 months**: (Purpose: Reminder that data can be shared with your HCP during any consultation):
   Kjære deltager. Husk at du kan dele data som du registrerer i Diabetesdagboka med helsepersonell uansett når du er til konsultasjon. Bare klikk på menyen oppe til høyre og velg «Del data».
4. Follow-up after **3 months**: (Purpose: Happy halfway + Set goals might be helpful, we have found that it helps ourselves?)
   Hei, har du prøvd å sette personlige mål i Diabetesdagboka? Du gjør dette ved å klikke på teksten «Hovedmål» øverst i app’en. Disse kan også være nyttig å diskutere med helsepersonell. Du kan lese om hvordan du setter mål i bruksanvisningen her (side 4): <https://fullflow.ehealthresearch.no/files/Diabetesdagboka/Bruksanvisning-for-Diabetesdagboka_20181031.pdf>
5. Follow-up after **4 months**: (Purpose: gathering a complete set of data even just for a short period, might be helpful when discussing your situation with your HCP)
   Kjære deltager. Vi vet at ikke alle registrerer data i app’en hele tiden, men det å samle inn mer data i forkant av en konsultasjon kan være nyttig, slik at man kan diskutere spesifikke utfordringer med din lege eller sykepleier under konsultasjonen.
6. Follow-up after **5 months**: (Purpose: Remember to scedule a consultation where you can share your data.)
   Hei, du har nå vært med i studien i rundt 5 måneder og det er tid for å bestille time for en konsultasjon hvor du kan dele og diskutere dine data. Dersom dette medfører ekstrautgifter for deg, kan du kontakte oss slik at du får refundert disse. E-post: [fullflow2019@ehealthresearch.no](mailto:fullflow2019@ehealthresearch.no)
7. Follow-up after **6 months**: (Purpose: Invitation to study-end focus group/co-design workshops, sent AFTER they’ve been the consultation)
   Tusen takk for at du deltok i vår studie! Vi vil gjerne invitere deg til en 2-timers workshop ved UNN/Forskningsparken i Tromsø på ettermiddagen den dag.måned.2019, klokka hh:mm. Der vil vi invitere deg til å dele dine erfaringer og ideer med oss forskere. Vi vil der diskutere dine erfaringer og ideer til hvordan egne innsamlede helsedata best kan deles med helsepersonell. Vi tilbyr reisekompensasjon. Send oss en e-post og meld deg på: [fullflow2019@ehealthresearch.no](mailto:fullflow2019@ehealthresearch.no)
8. Follow-up after **7-8** **months**: (Purpose: Thank you again for participating. Your contribution is very valuable for better understand how mobile technology can support you and your HCP)
   Vi har nå avsluttet vår studie og vil gjerne takke deg for din deltagelse! Din deltagelse er veldig nyttig for forståelse og utvikling av hvordan mobil helseteknologi kan støtte opp om deg og helsepersonell.
9. Catch-all message, offering our help: (Purpose: Follow-up if stopped using the Diabetesdagboka)
   Hei! Dersom du har spørsmål om de ulike måtene å bruke appen på, husk at vi kan hjelpe deg underveis i studien. Vi kan for eksempel gi deg en praktisk gjennomgang i bruken, eller mer informasjon. Du kan avtale et møte med oss på vårt kontor i Forskningsparken i Tromsø, snakke med oss over Skype eller sende oss e-post med spørsmål.
